# Space-time-coding metasurfaces for high-dimensional communications with OAM-, polarization-, and frequency-division multiplexing

**DOI:** 10.1038/s41377-026-02282-w

**Published:** 2026-04-20

**Authors:** Lei Zhang, Tie Jun Cui

**Affiliations:** State Key Laboratory of Millimeter Waves, School of Information Science and Engineering, Southeast University, Nanjing, China

**Keywords:** Metamaterials, Optical physics

## Abstract

Recent advances in high-dimensional multiplexing have enabled the concurrent operation of multiple independent communication channels through orbital angular momentum, polarization, and frequency division multiplexing, all implemented on a compact space-time-coding metasurface platform. These developments provide a streamlined and high-efficiency approach to optimizing multiplexing performance and enhancing channel capacity in wireless communication systems.

## Abstract (web summary)

Space-time-coding metasurfaces enable high-dimensional communications by concurrently operating multiple independent communication channels via orbital angular momentum, polarization, and frequency-division multiplexing.

## Graphical abstract

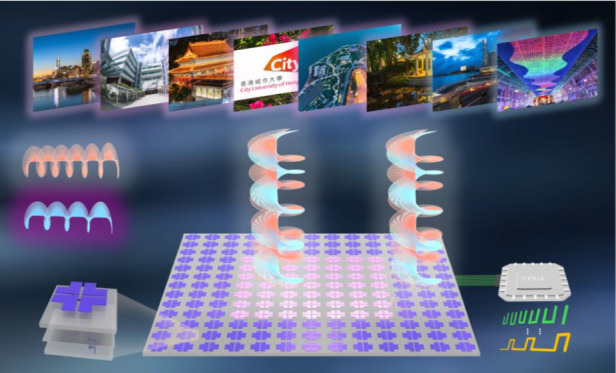

In wireless communication systems, multiplexing plays a pivotal role in the efficient utilization of limited spectral and spatial resources. Beyond the conventional multiplexing schemes operating in the frequency, polarization, and time domains—which are ubiquitously employed in state-of-the-art wireless communication systems—orbital angular momentum (OAM) multiplexing introduces a novel, orthogonal physical domain for multiplexing implementation^1^. However, OAM multiplexing requires precise manipulation of electromagnetic (EM) wavefields, which renders the hardware architectures reported in previous studies bulky, complex, and difficult to integrate.

A promising strategy to simplify the hardware platform for orbital angular momentum (OAM) generation and manipulation lies in the adoption of programmable metasurfaces^2^. By embedding programmable components into artificially engineered structures, such metasurfaces enable dynamic, time-varying control of EM wavefronts, making them particularly well-suited for versatile applications. To further enhance EM manipulation capabilities, Zhang et al. proposed space-time-coding metasurfaces (STCMs)^3,4^, introducing a novel paradigm that enables dynamic control of EM wave properties in both spatial and time domains. With the rapid development of STCMs, a variety of novel physical phenomena have been demonstrated across both microwave and optical frequency regimes^5–7^. To expand their application scope, extensive studies have explored the use of STCMs in new-architecture wireless communication^8–11^ and radar^12^ systems, integrated sensing and communication^13^, electronic countermeasures^14^, antenna design^15,16^, and dynamic holograms^17^. Nevertheless, most existing STCM-based communication studies remain primarily focused on space-frequency and/or polarization multiplexing techniques^8–10^, indicating that the full potential of STCMs for enhancing wireless communication capacity has yet to be fully exploited.

In a recent paper published in Light: Science & Applications, Geng-bo Wu’s research team at City University of Hong Kong proposed a high-dimensional multiplexing wireless communication system based on space-time-coding metasurfaces^18^. As illustrated in Fig. 1, by employing space-time-coding sequences and regional control of a dual-polarized programmable metasurface, the researchers simultaneously realized OAM-, polarization-, and frequency-multiplexing within a compact and structurally compact platform. The proposed system supports eight independent quadrature phase shift keying (QPSK) communication channels, achieving a signal-to-noise ratio (SNR) exceeding 12.5 dB and a low bit error rate (BER) of 10^−5^, thereby substantially increasing the communication capacity.

**Fig. 1:**
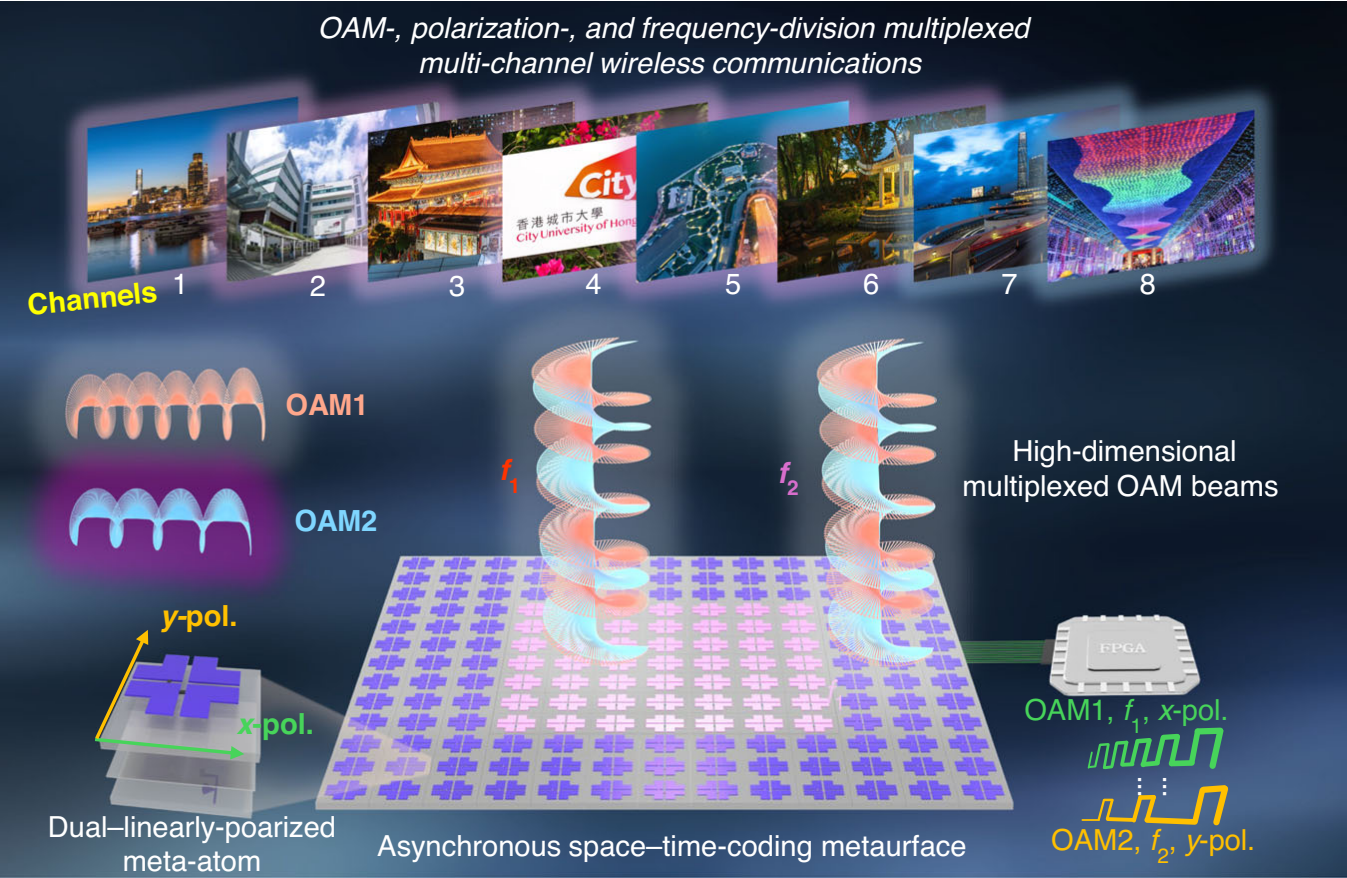
OAM-, polarization-, and frequency-division multiplexed multi-channel wireless communications based on an asynchronous space-time-coding metasurface

To meet the stringent requirements for precise and flexible EM wave control in high-dimensional multiplexing systems, a dual-polarized asynchronous space-time-coding metasurface (DASM) is introduced as a versatile hardware platform for realizing multi-channel wireless communications. In contrast to conventional OAM communication systems that rely on complex analog, digital, and radio-frequency components, the proposed DASM-based system enables direct modulation, frequency multiplexing, and OAM beam generation using a single programmable surface, significantly reducing hardware complexity. Owing to its dual-polarized programmability, the DASM allows independent manipulation of EM waves in orthogonal polarizations (*x*- and *y*-directions), thereby facilitating polarization multiplexing. Moreover, by applying distinct time-coding sequences, the reflection amplitude and phase at harmonic frequencies can be independently controlled, enabling the generation of superimposed states of multiple OAM modes and realizing OAM multiplexing. To support frequency multiplexing and suppress inter-channel interference, the aperture of the DASM is spatially partitioned into two distinct regions, each dedicated to time modulation and wave manipulation at different harmonic frequencies. Importantly, since the OAM, polarization, and frequency dimensions are mutually orthogonal, the total number of multiplexing channels in the proposed system equals the product of the number of channels in each individual dimension.

To experimentally validate the high-dimensional multiplexing capability, a prototype communication system was implemented using a 12×12 DASM array. The system was designed to simultaneously transmit independent data streams through eight independent multiplexing channels, incorporating orthogonal *x*- and *y*-polarizations, frequency-offset modulation at 250 kHz and 100 kHz, and OAM modes with *l* = ±2. At the receiver, an OAM demultiplexing lens, a dual-linearly polarized antenna, and a mixer were employed to separate signals differing in OAM mode, polarization, and frequency. Measurement results demonstrate successful demultiplexing of the received signals with negligible inter-channel crosstalk, thereby confirming the feasibility and efficiency of the proposed architecture.

Overall, the proposed high-dimensional multiplexing wireless communication system enables low-cost, high-capacity data transmission by fully exploiting limited EM spectrum and hardware resources, which represents a significant advancement in vortex EM waves manipulation and multiplexing. Furthermore, the integration of non-diffracting beam control techniques could further extend the system’s applicability from short-range to long-range communication scenarios. Although the number of OAM multiplexing layers and the achievable symbol rate are constrained by the metasurface array scale and the switching speed of PIN diodes, the proposed system still exhibits considerable potential for practical wireless communication applications.

## References

[1] Wang, J. et al. Terabit free-space data transmission employing orbital angular momentum multiplexing. *Nat. Photonics* **6**, 488–496 (2012).

[2] Cui, T. J. et al. Coding metamaterials, digital metamaterials and programmable metamaterials. *Light Sci. Appl.* **3**, e218 (2014).

[3] Zhang, L. et al. Space-time-coding digital metasurfaces. *Nat. Commun.* **9**, 4334 (2018). 30337522 10.1038/s41467-018-06802-0 PMC6194064

[4] Zhang, L. & Cui, T. J. Space-time-coding digital metasurfaces: principles and applications. *Research* **2021**, 9802673 (2021). 34386772 10.34133/2021/9802673 PMC8328401

[5] Cardin, A. E. et al. Surface-wave-assisted nonreciprocity in spatio-temporally modulated metasurfaces. *Nat. Commun.* **11**, 1469 (2020). 32193393 10.1038/s41467-020-15273-1 PMC7081213

[6] Wang, S. R. et al. Asynchronous space-time-coding digital metasurface. *Adv. Sci.* **9**, 2200106 (2022). 10.1002/advs.202200106 PMC9405512 35751468

[7] Sisler, J. et al. Electrically tunable space-time metasurfaces at optical frequencies. *Nat. Nanotechnol.* **19**, 1491–1498 (2024). 39048705 10.1038/s41565-024-01728-9

[8] Zhang, L. et al. A wireless communication scheme based on space- and frequency-division multiplexing using digital metasurfaces. *Nat. Electron.* **4**, 218–227 (2021).

[9] Ke, J. C. et al. Space-frequency-polarization-division multiplexed wireless communication system using anisotropic space-time-coding digital metasurface. *Natl. Sci. Rev.* **9**, nwac225 (2022). 36452428 10.1093/nsr/nwac225 PMC9701098

[10] Wang, S. R. et al. Manipulations of multi-frequency waves and signals via multi-partition asynchronous space-time-coding digital metasurface. *Nat. Commun.* **14**, 5377 (2023). 37666804 10.1038/s41467-023-41031-0 PMC10477258

[11] Wang, L. et al. High-order direct modulation terahertz communications with a wideband time-coding metachip modulator. *Sci. Adv.* **10**, eadq8693 (2024). 39576862 10.1126/sciadv.adq8693 PMC11801051

[12] Wang, S. R. et al. Simplified radar architecture based on information metasurface. *Nat. Commun.* **16**, 6505 (2025). 40664680 10.1038/s41467-025-61934-4 PMC12263977

[13] Chen, X. Q. et al. Integrated sensing and communication based on space-time-coding metasurfaces. *Nat. Commun.* **16**, 1836 (2025). 39984472 10.1038/s41467-025-57137-6 PMC11845482

[14] Sun, Z. Z. et al. Anti-radar based on metasurface. *Nat. Commun.* **16**, 7258 (2025). 40770186 10.1038/s41467-025-62633-w PMC12328761

[15] Wu, G. B. et al. Sideband-free space–time-coding metasurface antennas. *Nat. Electron.* **5**, 808–819 (2022).

[16] Wu, G. B. et al. A universal metasurface antenna to manipulate all fundamental characteristics of electromagnetic waves. *Nat. Commun.* **14**, 5155 (2023). 37620303 10.1038/s41467-023-40717-9 PMC10449906

[17] Wu, G. B. et al. A space-time holographic metasurface antenna. *Sci. Adv.* **11**, eadx7090 (2025). 40961192 10.1126/sciadv.adx7090 PMC13155563

[18] Yang, C. F. et al. High-dimensional multiplexing through vortex electromagnetic wave manipulation by space-time-coding metasurfaces. *Light Sci. Appl.* **15**, 160 (2026). 41796115 10.1038/s41377-026-02232-6 PMC12968085

